# High-Resolution Mass Spectrometry Reveals Distinct Temporal Accumulation Patterns of Metabolites in Reproductive Organs of Purple- and White-Flowered *Platycodon grandiflorus* Across Developmental Stages

**DOI:** 10.3390/life16081260

**Published:** 2026-07-30

**Authors:** Jun Lao, Nannan Wang, Chuyu Yao, Xiangmin Piao

**Affiliations:** 1 College of Biology and Food Engineering, Jilin University of Chemical Technology, Jilin 132022, China; wangnannan1@jlict.edu.cn; 2 College of Chinese Medicinal Materials, Jilin Agricultural University, Changchun 130118, China; yaochuyu1020@163.com (C.Y.); piaoxiangmin@jlau.edu.cn (X.P.)

**Keywords:** *Platycodon grandiflorus*, high-resolution mass spectrometry, Mfuzz, metabolites, reproductive organs

## Abstract

Background: Flower color is a well-defined trait in *Platycodon grandiflorus,* whereas little is known about the effects of flower color variation on the metabolic profiles of reproductive organs. Triterpenoid saponins and flavonoids have common precursors upstream, suggesting a potential carbon flux trade-off. Methods: Untargeted UPLC-MS/MS metabolomics was performed on the reproductive organs of purple- and white-flowered *P. grandiflorus* at six stages of flower development. Mfuzz time-series clustering, PCA, and metabolite correlation networks were used for data analysis. Results: Six clusters were assigned to 25 metabolites (17 triterpenoid saponins and 8 flavonoids). Flavonoid glycosides were found to possess a conserved inverted V-shaped accumulation pattern in both germplasms. By contrast, saponins derived from triterpenoids showed strong germplasm-dependent accumulation patterns. White-flowered plants showed sustained accumulation, with a peak at the young fruit stage. In purple-flowered plants, accumulation peaked transiently at the withering stage, followed by a decline. PCA validated that metabolic divergence increased with developmental progression. Conclusions: Based on the metabolomic profiles, we hypothesize a putative trade-off model in which floral color divergence may change upstream carbon flux allocation between the triterpenoid saponin and flavonoid pathways. The young fruit stage of white-flowered plants is a candidate harvesting period for bioactive saponins. These conclusions are based only on the pattern of metabolite accumulation and need to be validated by multi-omics.

## 1. Introduction

*Platycodon grandiflorus* is a perennial herbaceous plant belonging to the Campanulaceae family [1]. It is used medicinally in East Asia for traditional applications such as dispersing the lung [2], relieving sore throat, expelling phlegm [3], and draining pus. Triterpenoid saponins are the main bioactive components of *P. grandiflorus*, possessing antitussive, expectorant, anti-inflammatory [4], antioxidant [5,6], anti-tumor [7,8], and hepatoprotective effects [9,10]. Through long-term natural selection and artificial domestication, *P. grandiflorus* has acquired substantial intraspecific genetic diversity, with flower color variation being the most distinguishable phenotypic trait. Cultivated populations are mostly classified into purple-flowered and white-flowered types [11]. Because petal tissue is directly responsible for flower color, the levels of secondary metabolites (anthocyanins, flavonoids, and triterpenoid saponins) in reproductive organs are closely related to both pigmentation and medicinal quality [12].

Metabolic studies on *P. grandiflorus* have primarily focused on root tissues. Kwon et al. [13] characterized platycodin D content variation across regions and processing methods; Matsuda et al. [14] measured seasonal saponin dynamics; Liu et al. [15] compared extraction methods; and UPLC-QTOF-MSE analysis confirmed the roots as the main site of saponin accumulation [16]. Taken together, these findings indicate that systematic analyses of floral secondary metabolites remain limited.

Several important questions remain unanswered. Temporal metabolic changes in reproductive organs (buds, corollas, and young fruits) have not been systematically studied. As a herb that reproduces mostly by seed, *P. grandiflorus* relies heavily on its reproductive organs for seed quality. Although purple- and white-flowered strains differ in morphology and metabolite accumulation, no systematic comparison of the global metabolic profiles of reproductive organs has been established. Saponin-enriched plant extracts exhibit dual agricultural functions, including broad-spectrum antifungal activity and improved seed germination under stress conditions [17]. *P. grandiflorus* accumulates abundant triterpenoid saponins in its reproductive organs, and elucidating the dynamic metabolic differentiation between white- and purple-flowered germplasms could inform the agricultural utilization of these floral tissues.

It remains unclear how the accumulation of key secondary metabolites (triterpenoid saponins and flavonoids) differs across developmental stages. Triterpenoid saponins and flavonoids share upstream precursors from the MVA and MEP pathways (isopentenyl pyrophosphate and dimethylallyl pyrophosphate), and silencing the F3′5′H gene in white-flowered plants disrupts anthocyanin synthesis [18]. An unresolved question is whether this upstream flux blockage creates a temporal trade-off or metabolic reallocation between the triterpenoid saponin and flavonoid pathways in reproductive organs.

We applied UPLC-MS/MS-based untargeted metabolomics to qualitatively and quantitatively profile secondary metabolites in the reproductive organs (from the young bud to the young fruit stage, covering six developmental stages) of purple-flowered and white-flowered *P. grandiflorus.* Mfuzz time-series clustering, PCA, differential metabolite screening, and metabolite correlation networks were used to (i) characterize global metabolic differences between the two color types; (ii) identify key temporally differential metabolites; and (iii) elucidate competitive and synergistic relationships between the triterpenoid saponin and flavonoid pathways. These findings are intended to provide a metabolomic basis for understanding flower-color-related metabolic divergence in reproductive organs. Notably, this study is based solely on metabolite accumulation patterns without synchronous transcriptomic profiling or enzyme activity quantification; the proposed carbon flux hypothesis requires multi-omics validation.

## 2. Materials and Methods

### 2.1. Plant Materials

All purple-flowered and white-flowered *Platycodon grandiflorus* germplasms used in this study are artificially screened strains maintained in the medicinal herb garden of Jilin Agricultural University. The two germplasm lines are preserved only in our research group’s on-campus planting plot for laboratory research and have not been archived in a formal standardized germplasm resource bank; therefore, no official germplasm accession numbers are available. Morphological identification of the two flower-color variants was completed by Dr. Xiangmin Piao. Healthy and vigorously growing reproductive organs were collected at six stages: Time 1 (young bud stage), Time 2 (medium bud stage), Time 3 (pre-flowering stage), Time 4 (full blooming stage), Time 5 (post-flowering withering stage), and Time 6 (young fruit stage). Fresh samples were immediately snap-frozen in liquid nitrogen to prevent saponin degradation and then stored in an ultra-low-temperature freezer at −80 °C.

### 2.2. Sample Preparation and UPLC-MS/MS Analysis

Methanol and acetonitrile (HPLC-grade), formic acid (MS-grade), and reference standards of platycodin D and platycodin D3 (purity > 98%, Shanghai Yuanye Bio-Technology Co., Shanghai, China) were used. Freeze-dried samples were ground and passed through a standard 40-mesh sieve. Powder (0.5 g) was placed in a 50 mL centrifuge tube, and 25 mL of methanol was added as the solvent. Ultrasonic extraction was performed at room temperature (100 W; 40 kHz) for 30 min. After extraction, the supernatant was collected, filtered through a 0.22 μm organic membrane filter, and transferred to a liquid chromatography vial. The column temperature was maintained at 35 °C. The mobile phase consisted of 0.1% formic acid in water (phase A) and pure acetonitrile (phase B). The flow rate was set at 0.3 mL/min, and the injection volume was 1 μL. Mass spectrometry was performed using a Thermo Fisher (Waltham, MA, USA) Orbitrap Tribrid mass spectrometer in negative electrospray ionization mode at a spray voltage of 2700 V. For each sample, full-scan or DDA secondary fragmentation scans were performed simultaneously for metabolite qualitative identification and relative quantification. Ion source parameters: sheath gas flow rate, 40 Arb; auxiliary gas flow rate, 5 Arb; ion transfer tube temperature, 320 °C; vaporizer temperature, 320 °C; mass scan range, 350–1500; HCD collision energy, 40%; Orbitrap full-scan resolution, 60,000.

### 2.3. Data Processing and Statistical Analysis

Raw mass spectrometry data were processed using Xcalibur software (version 3.1). Metabolites were annotated based on the retention time, precursor *m*/*z*, and MS/MS fragmentation spectra. Time-series fuzzy clustering was performed using the Mfuzz package [19] in R 4.5.2 with six clusters. OPLS-DA was used to maximize group separation; metabolites with VIP > 1.0, *p* < 0.05 (*t*-test), and BH-FDR-adjusted *p* < 0.05 were considered significant. PCA was used to visualize the overall metabolic profiles. OPLS-DA models were validated via 200 permutation tests (R^2^Y and Q^2^ are provided in the Supplementary Materials). Correlation networks were constructed using Pearson correlation (|r| > 0.55, *p* < 0.05) via the Hmisc package, split into mid (Time 2–4) and late (Time 5–6) stages. Integrating these results with Mfuzz clustering, we analyzed the triterpenoid saponin biosynthetic pathway across developmental stages and floral color types. Effect sizes and post hoc power were not calculated in the present study. As an exploratory untargeted metabolomics study with a limited sample size (*n* = 3 per group) and a large number of quantified metabolites, we prioritized stringent FDR correction to minimize false positives, which is the standard approach in exploratory metabolomics.

## 3. Results

### 3.1. Overview of Mfuzz Time-Series Clustering of Metabolites

Twenty-five stably quantifiable secondary metabolite monomers were detected and annotated (Table 1 and Table S1). Based on Mfuzz time-series clustering, six characteristic temporal expression patterns (Clusters 1 to 6) were identified. The metabolite composition of each cluster is summarized in Table 1 and comprises triterpenoid saponins (protosaponins, acetylated saponins, and Acetyl-m12 isomers), isorhamnetin-derived flavonoid glycosides, and polyacetylene glycosides.

### 3.2. Temporal Expression Patterns and Monomer Composition Characteristics of the Six Clusters

**Figure 1:**
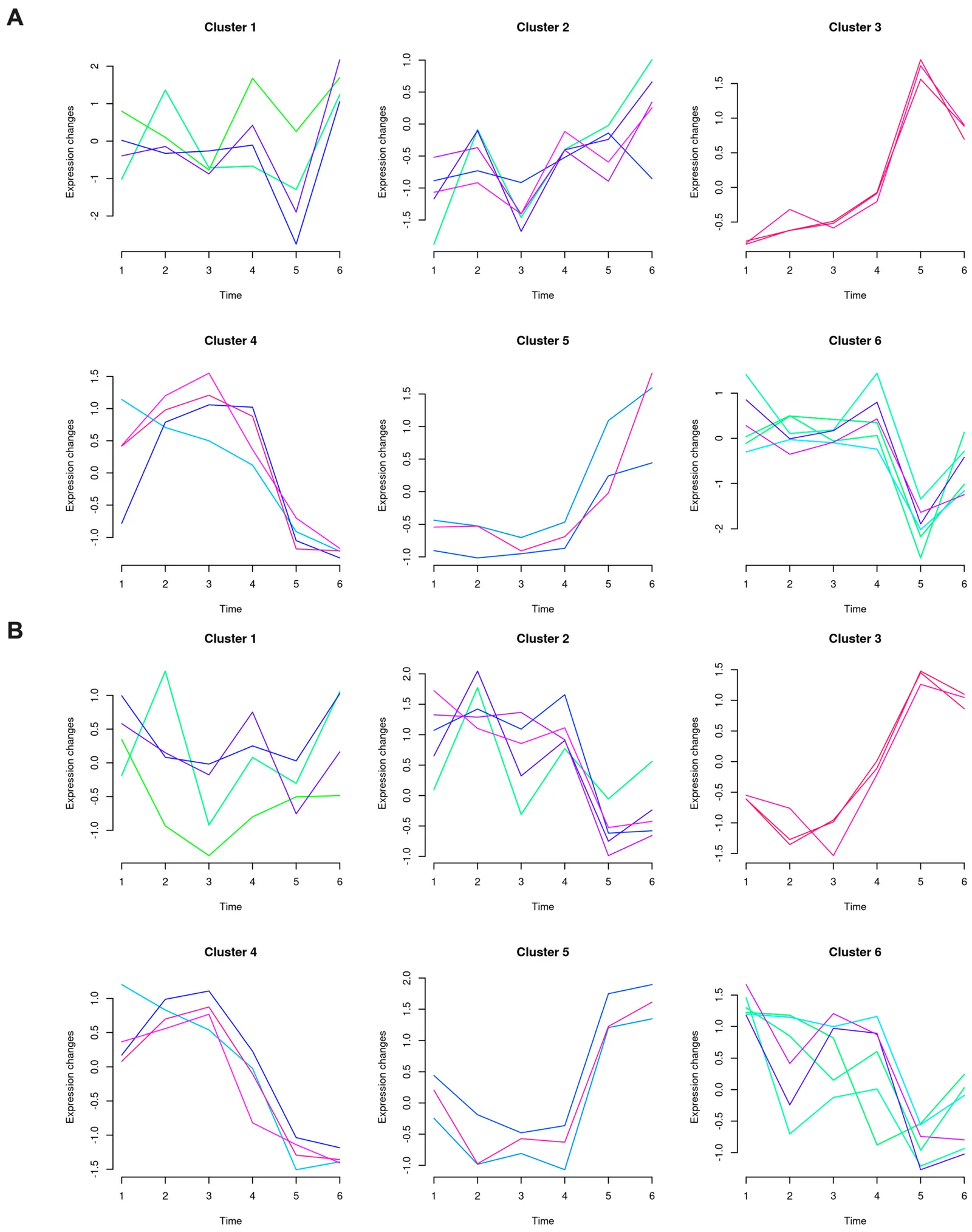
Dynamic changes in metabolites in purple-flowered and white-flowered *P. grandiflorus* at different developmental stages. (**A**) Dynamic metabolite changes in the reproductive organs of white-flowered *P. grandiflorus*. (**B**) Dynamic metabolite changes in the reproductive organs of purple-flowered *P. grandifloras*. All visualization graphs were generated using R software v4.5.2. Three independent biological replicates were set for each group, and the mean values were calculated.

Time-series fitting analyses were performed on the metabolites of purple-flowered and white-flowered *P. grandiflorus*. The accumulation dynamics of the six clusters in the reproductive organs across the developmental cycle are shown in Figure 1.

Among the six clusters, Clusters 1–3 and 5–6 were predominantly composed of triterpenoid saponins (including protosaponins, acetylated saponins, and acetyl-m12 isomers). Despite their compositional diversity, these saponin clusters shared a common germplasm-dependent pattern: white-flowered *P. grandiflorus* consistently exhibited late-stage accumulation (peaking at the young fruit stage, Time 6), whereas purple-flowered *P. grandiflorus* showed mid-stage peaks or oscillatory patterns with a decline at the young fruit stage. This reversed differentiation was most pronounced in Clusters 2 and 5 (‘completely opposite’ patterns) and Cluster 3 (acetyl-m12 isomers: linear rise in white vs. oscillatory in purple). Cluster 1 acetylated saponins showed a fluctuating bimodal pattern in white-flowered plants, with high-expression windows at the full blooming and young fruit stages, while purple-flowered plants concentrated expression at the early and terminal stages.

Cluster 4 comprised exclusively isorhamnetin-derived flavonoid glycosides. Unlike the saponin clusters, these compounds exhibited a conserved inverted V-shaped pattern with mid-stage peaks in both germplasms, indicating that flavonoid glycoside biosynthesis is regulated independently of floral color variation. Cluster 5 contained a mixture of triterpenoid saponins, taxifolin, and lobetyolin, with white-flowered plants showing a continuous linear increase to a global maximum at young fruit, while purple-flowered plants exhibited a V-shaped pattern with early and late peaks.

Cluster 6, containing the pharmacopeia-indicative compounds platycodin D and platycodin J, mirrored the overall saponin trend. The most striking germplasm divergence was observed in Cluster 3 (acetyl-m12 series): white-flowered plants accumulated all three isomers linearly and continuously from young buds to young fruit, while purple-flowered plants showed a compressed mid-stage peak followed by a rapid decline, representing one of the most defining metabolic phenotypic differences between the two germplasms.

### 3.3. Global Metabolic Temporal Characteristics Revealed by Mfuzz Clustering Heatmap

**Figure 2:**
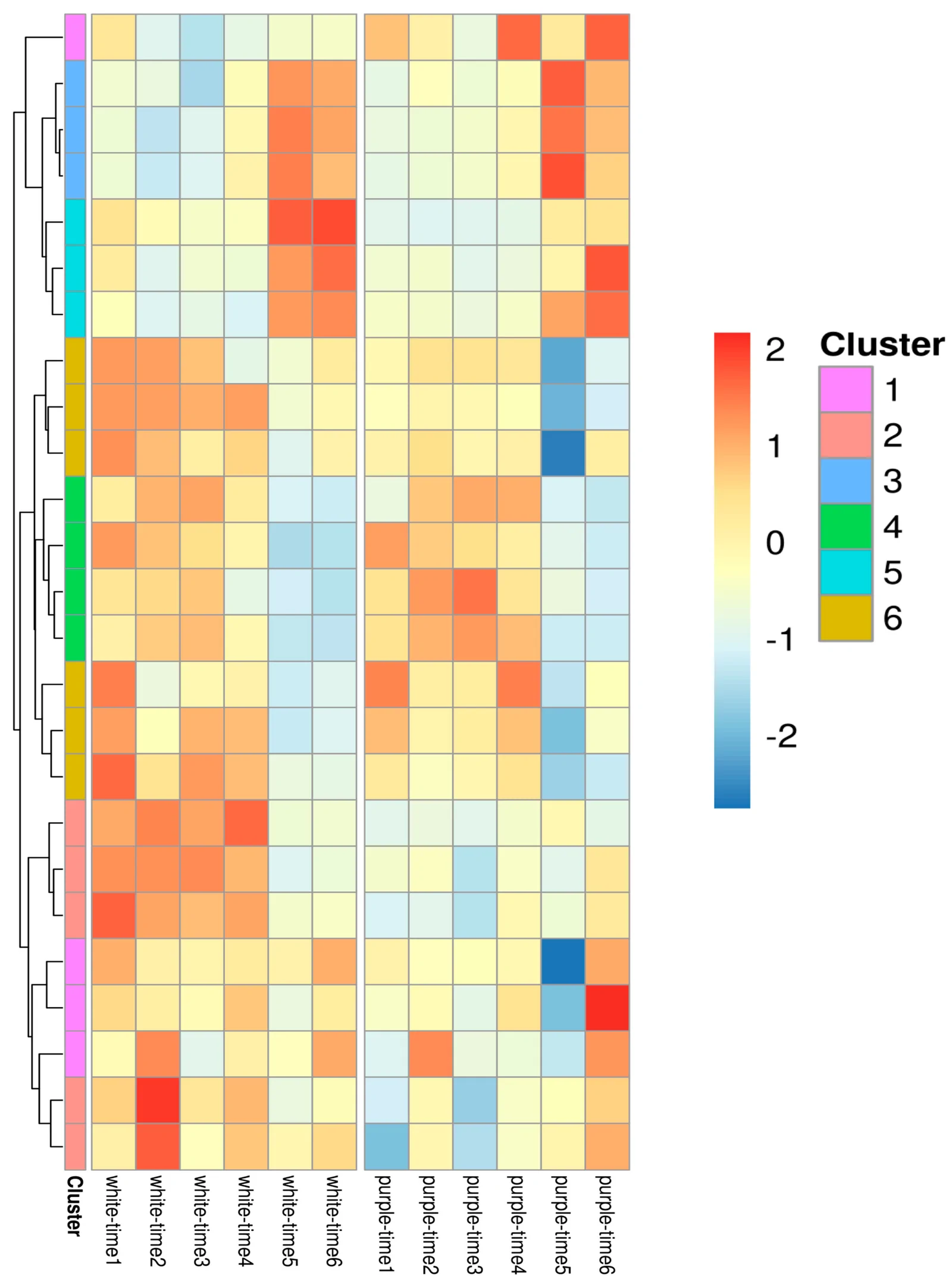
Heatmap of distinct metabolite clusters in reproductive organs of purple-flowered and white-flowered *Platycodon grandiflorus*. All visualization graphs were generated using R software v4.5.2; three independent biological replicates (*n* = 3) were set for each sampling group.

The Mfuzz clustering heatmap (Figure 2) confirmed that the spatiotemporal accumulation patterns were highly germplasm-dependent. White-flowered *P. grandiflorus* showed high-abundance blocks spanning from full blooming (Time 4) to young fruit (Time 6), indicating a synchronized late-stage enrichment. In purple-flowered *P. grandiflorus*, most metabolites remained low from young bud (Time 1) to full blooming (Time 4), with orange-red blocks appearing only at withering and young fruit stages (Time 5–6). The time color-block variation patterns were broadly similar among the metabolites within each cluster, while the high-expression windows and accumulation abundances differed markedly across clusters, with clearly defined cluster boundaries confirming the biological discriminative power of the six-cluster model. Saponins followed a delayed accumulation pattern typical of maturation-stage secondary metabolites.

Based on the temporal patterns, the secondary metabolic process can be summarized as three phases: (1) Young bud stage (Time 1): all saponin clusters showed low expression, with only a few metabolites in Cluster 5 beginning biosynthesis. (2) Mid-development (Time 2–4): flavonoid glycosides (Cluster 4) showed high abundance, capturing most of the carbon flux. In white-flowered plants, saponin biosynthesis in Clusters 1, 3, and 6 was concurrently activated. (3) Late stage (Time 5–6): flavonoid signal decreased, and saponins in Clusters 1, 2, and 6 were synchronously upregulated in a burst-like manner, especially in white-flowered plants, while purple-flowered plants showed only a transient peak at withering followed by sharp decline at young fruit, indicating that purple-flowered plants lack the sustained saponin accumulation capacity observed in white-flowered plants.

### 3.4. Principal Component Analysis of Differential Metabolites Between the Two Germplasms at Mid- and Late Developmental Stages

**Figure 3:**
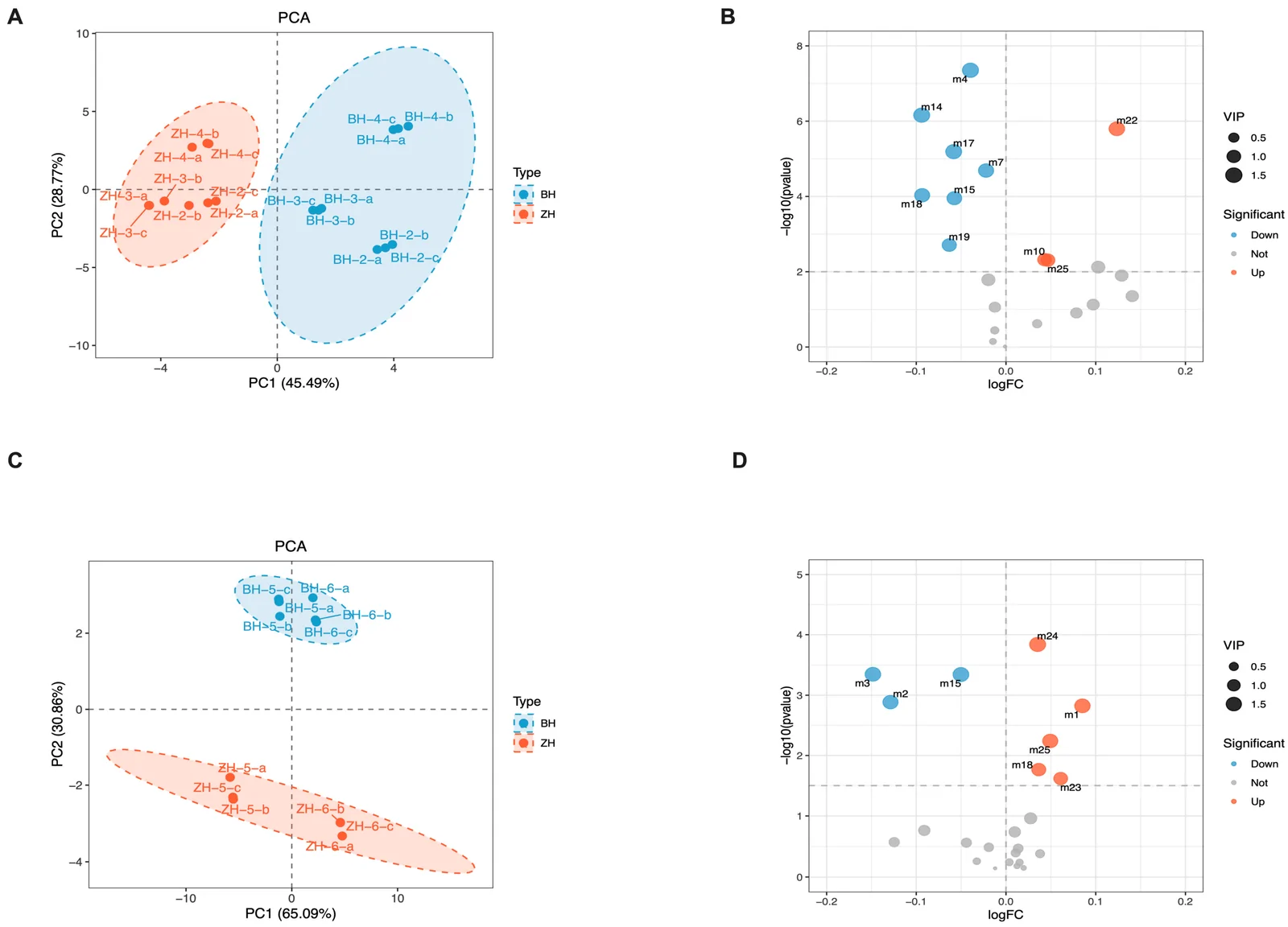
PCA and volcano plots of differential metabolites between purple-flowered (ZH) and white-flowered (BH) *P. grandiflorus* at the mid-development (Time 2–4) and late-development (Time 5–6) stages. (**A**,**C**) PCA score plots; (**B**,**D**) volcano plots for the corresponding stages. BH, white-flowered; ZH, purple-flowered; a–c, biological replicates. R software v4.5.2 (*n* = 3).

Based on the Mfuzz clustering results, PCA was performed on mid-development (Time 2–4) and late-development (Time 5–6) samples (Figure 3).

At mid-development (Time 2–4), PC1 and PC2 accounted for 45.49% and 28.77% of the variance, respectively (cumulative 74.26%). Purple- and white-flowered samples separated along PC1 without overlap, indicating that floral color was the main driver of metabolic differentiation at this stage. Biological replicates clustered closely, confirming experimental reproducibility. Samples from Time 2, Time 3, and Time 4 showed a gradient distribution along PC2, indicating that developmental progression also shaped the metabolic profile even within the mid-developmental window. Protosaponins and acetylated saponins (platycodin D, acetyl-platycodin D, platycodin L, and 3″-O-acetyl-polygalacin D3) were upregulated in white-flowered plants, along with flavonoid glycoside monomers (m4 and m22), suggesting active competition for carbon flux between the two pathways.

At the terminal stage (Time 5–6), PC1 and PC2 represented 65.09% and 30.86% (cumulative 95.95%). The separation between germplasms was greater than that at mid-development, confirming that floral-color-mediated metabolic divergence intensified with organ maturation. Biological replicates of the same germplasm clustered very tightly, confirming the stability and reliability of the experiment. Samples from Time 5 and Time 6 showed a weaker gradient distribution along PC2, confirming that dynamic metabolic changes persisted even at fully mature organs. Many highly differential metabolite monomers (m1, m2, m3, m15, m18, m23, m24, and m25) still existed between the two germplasms at the terminal stage. The upregulation and downregulation patterns of differential metabolites at this stage differed markedly from mid-development, confirming that the metabolic divergence patterns established at mid-development continued to evolve as the organs matured.

### 3.5. Time-Series Metabolite Correlation Networks Show Floral-Color-Mediated Metabolic Reconfiguration

**Figure 4:**
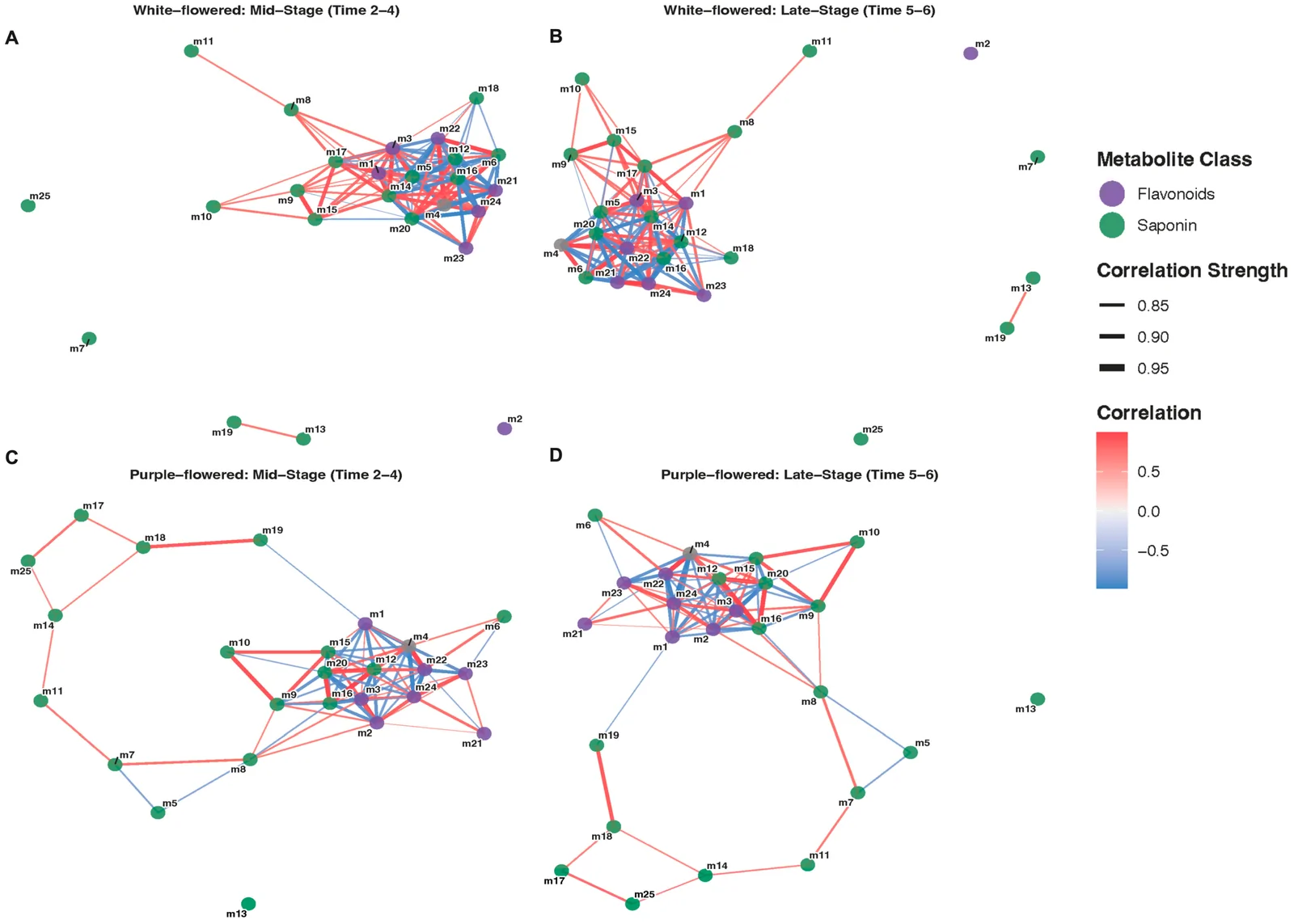
Time-series metabolite correlation networks between purple- and white-flowered *P. grandiflorus* in early and late development. (**A**,**B**) metabolite correlation networks of White-flowered *P. grandiflorus* in Mid-Stage and Late-Stage; (**C**,**D**) metabolite correlation networks of Purple-flowered *P. grandiflorus* in Mid-Stage and Late-Stage;Nodes represent different metabolites; node colors: metabolites (purple: flavonoid glycosides; green: triterpenoid saponins; gray: others). Node sizes are uniform; edge thickness represents absolute Pearson correlation coefficient (|r|); and edge color represents direction of correlation (red: positive; blue: negative). All visualization graphs were generated using R software v4.5.2; three independent biological replicates (*n* = 3) were set for each sampling group.

Time-sliced correlation networks were constructed for mid (Time 2–4) and late (Time 5–6) developmental stages (Figure 4). Node colors represent metabolite classes (purple: flavonoid glycosides; green: triterpenoid saponins); edge thickness indicates absolute Pearson |r|, and edge color indicates correlation direction (red: positive; blue: negative).

At mid-development, the networks of both germplasms were symmetrically joined, with flavonoid and saponin nodes interspersed at moderate edge thickness and mixed red/blue connections, suggesting active substrate competition without a dominant module. At late development, the networks diverged markedly, consistent with the increasing metabolic separation reflected by PCA (PC1 contribution rising from 45.49% to 65.09%). White-flowered *P. grandiflorus* condensed into a tightly correlated triterpenoid saponin module anchored by m10 (platycodin D), with thickened edges connecting m9, m16, and m12 (acetylated saponins), indicating coordinated saponin biosynthesis at the young fruit stage. In contrast, the purple-flowered network became chaotic with thin, mixed red/blue edges and no modular structure, suggesting metabolic divergence at the young fruit stage due to limited precursor supply or degradation [22].

## 4. Discussion

### 4.1. Floral Color Variation Reshapes the Spatiotemporal Landscape of Secondary Metabolism in Reproductive Organs

Flower color is typically determined by the accumulation of delphinidin-type anthocyanins; the white flower color in most of these species is the result of the mutation or suppression of genes in the anthocyanin biosynthesis pathway [23]. Tanaka et al. [23] found flavonoid 3′5′H to be the key rate-limiting enzyme in the conversion of dihydrokaempferol to dihydromyricetin. In *P. grandiflorus*, Lv et al. [18] demonstrated through comparative transcriptomics that F3′5′H expression was extremely low (FPKM = 0.26) in white-flowered plants compared to 208.85 in purple-flowered plants, directly explaining the failure of white-flowered plants to accumulate delphinidin.

However, F3′5′H silencing does not results in the absolute loss of pigmentation. All of our Mfuzz clustering, PCA, and differential metabolite analyses point to triterpenoid saponins having robust germplasm-specific accumulation dynamics, whereas flavonoid glycosides held a conserved pattern. White-flowered plants were shown to always have saponin enrichment at late times, while purple-flowered plants concentrated the saponin accumulation earlier in development, and hence seem to shift the developmental time course of triterpenoid saponin biosynthesis.

From the perspective of the pathway, anthocyanins and triterpene saponin share precursors in the upper part (IPP and DMAPP from the MVA/MEP pathways). The purple-flowered plants channel the largest amount of carbon into the flavonoid–anthocyanin pathway at the middle of development, which competes with saponin biosynthesis. The saponins of purple-flowered plants only achieve a compensation-level peak under the condition of flavonoid pathway activity from the pre-flowering to withering stage. White-flowered plants, due to the lack of F3′5′H expression, relieve this competition and shunt excess carbon skeletons towards triterpenoid saponin biosynthesis and downstream glycosylation/acetylation modifications, which provides a possible explanation for the continuous linear accumulation of acetylated saponins (e.g., acetyl-m12 and acetyl-platycodin D) from the young bud to young fruit stage.

### 4.2. Metabolic Network Topology Reveals Floral-Color-Driven Metabolic Reconfiguration

The topological divergence between white- and purple-flowered networks at late development is consistent with a reconfiguration of carbon flux allocation mediated by the differential activity of downstream modifying enzymes.

White-flowered *P. grandiflorus* formed a tightly coordinated saponin module at the young fruit stage, with strongly positive correlations among platycodin D (m10), platycodin D3 (m9), and the acetyl-m12 series (m16/m12). This pattern is consistent with the enzymatic properties of UGT94BY1 [24], a broad-spectrum glycosyltransferase capable of the sequential glycosylation of triterpenoid saponins. With sufficient UDP-glucose and precursors, UGT94BY1 efficiently accelerates the conversion from platycodin D to platycodin D3, amplifying saponin accumulation in white-flowered plants.

In contrast, purple-flowered *P. grandiflorus* exhibited a disordered late-stage network with thin, mixed-correlation edges and no modular structure. This may result from the activation of glycoside hydrolases during tissue senescence, disrupting the terminal 6-OH group required for UGT94BY1-mediated chain extension [23]. The contrast between the highly condensed white-flowered module and the disorganized purple-flowered network provides compelling topological evidence that floral color variation may drive carbon flux reallocation.

### 4.3. Genomic and Enzymatic Bases of Triterpenoid Saponin Biosynthesis: The CYP450 Family Expansion and Downstream Glycosylation

The family expansion and UGT94BY1 glycosyltransferase regulatory mechanism described in this section are derived from previously published *Platycodon grandiflorus* genomic research rather than experimental data obtained in the present metabolomic study. Recent genomic advances in *P. grandiflorus* have shown that the mechanisms for triterpenoid saponin biosynthesis are very important. Yu et al. [25] (2025) reported the first T2T gap-free genome of *P. grandiflorus* and identified gene families involved in saponin skeleton biosynthesis and modification. They demonstrated that gene duplication (especially tandem and proximal duplications) is the main driving force for the expansion of the CYP450 family. They identified candidate CYP genes positively associated with platycodin accumulation, including three PgCYP716A, seven PgCYP72A, and seven PgCYP749A genes responsible for hydroxylation and oxidation modifications at critical positions (C-2, C-16, C-23, C-28, and C-24) of the triterpenoid skeleton, which determine the final types of platycodins.

Our time series metabolomics results are consistent with these results. White plant Clusters 2 and 6 had high accumulation peaks at the young fruit stage (Time 6). Based on Yu et al. [25], we hypothesize that white plants possess enough MVA pathway precursors at late development as well as more active or longer expression of CYP716A and CYP72A gene families responsible for downstream modifications. In particular, the specific spike in acetylated saponins (acetyl-m12 series, Cluster 3) in white plants is likely associated with the stage-specific activation of AT genes.

Recent work by Jiang et al. [24] showed a broad-spectrum, multi-step glycosyltransferase UGT94BY1 from *P. grandiflorus* that forms -(1,6) glycosidic bonds. This enzyme exhibits substrate promiscuity and is capable of continuously glycosylating the triterpenoid saponins (up to 3 glucose units) and flavonoid glycosides. Crucially, UGT94BY1’s catalytic activity depends on the free 6-OH group at the glucose moiety of the sugar acceptor.

The topological results observed in our networks (Figure 4B) are in accordance with the enzymatic property of UGT94BY1; the thick edges among m10-m9 and m10-m16/m12 exactly correspond to the two consecutive glycosylation reactions carried out by UGT94BY1. With sufficient UDP-glucose and precursors, UGT94BY1 effectively catalyzes the conversion from platycodin D (m10) to platycodin D3 (m9) in white-flowered plants at the young fruit stage. The thinner, chaotic edges in the purple-flowered late-stage network (Figure 4D) could be a consequence of the activation of glycoside hydrolases during tissue senescence that disrupt/block the terminus 6-OH group and end UGT94BY1 chain extension, adding to the disordered metabolic network of purple-flowered plants.

Thus, the upstream precursor release and downstream UGT94BY1-mediated enzymatic amplification combine to produce the coordinated saponin accumulation found in white-flowered plants, whereas the loss of the terminal recognition site for sugar chain elongation causes the disordered metabolic network in purple-flowered plants.

### 4.4. Reproductive Organs as a Novel Medicinal Resource

The traditional medicinal use of *P. grandiflorus* is mainly based on dried roots, and the Chinese Pharmacopeia still recommends peeling during processing. Chang et al. [26] (2021) used untargeted metabolomics (UPLC-Q-TOF/MS) to demonstrate that *P. grandiflorus* roots contain active triterpenoid saponins, suggesting that peeling can lead to the loss of bioactive compounds.

Building on these observations, we extend our results from medicinal components in roots to those in reproductive organs. The young fruit stage (Time 6) of white-flowered plants represents not only the global peak of saponin accumulation in the Mfuzz clustering analysis, but also the maximal metabolic synergy of the saponin module (platycodin D). Based on UGT94BY1, the white-flowered young fruit stage offers enough UDP-glucose donors and intact terminal 6-OH groups, enabling efficient sugar chain extension and accumulation. The metabolites at this stage are comparable to or even surpass those in early-stage roots. This suggests that the reproductive organs of white-flowered plants (especially from the withering to the young fruit stage) could be a promising non-medicinal resource for extracting triterpenoid saponins, especially highly bioactive acetylated forms. The specific enrichment of isorhamnetin-type flavonoid glycosides (Cluster 4) at the Full Blooming stage (Time 4) in purple-flowered plants provides a precise harvesting window for the targeted extraction of flavonoid antioxidants.

It should be noted that only three independent biological replicates were set for each group in the present exploratory metabolomics analysis. Such a limited sample size may restrict the generalizability of the observed metabolic differentiation patterns between the two floral germplasms. Multi-location field trials with expanded biological replicates and multi-year sampling are required in future work to further validate the universality of the carbon flux trade-off trend observed in this study.

## 5. Conclusions and Perspectives

**Figure 5:**
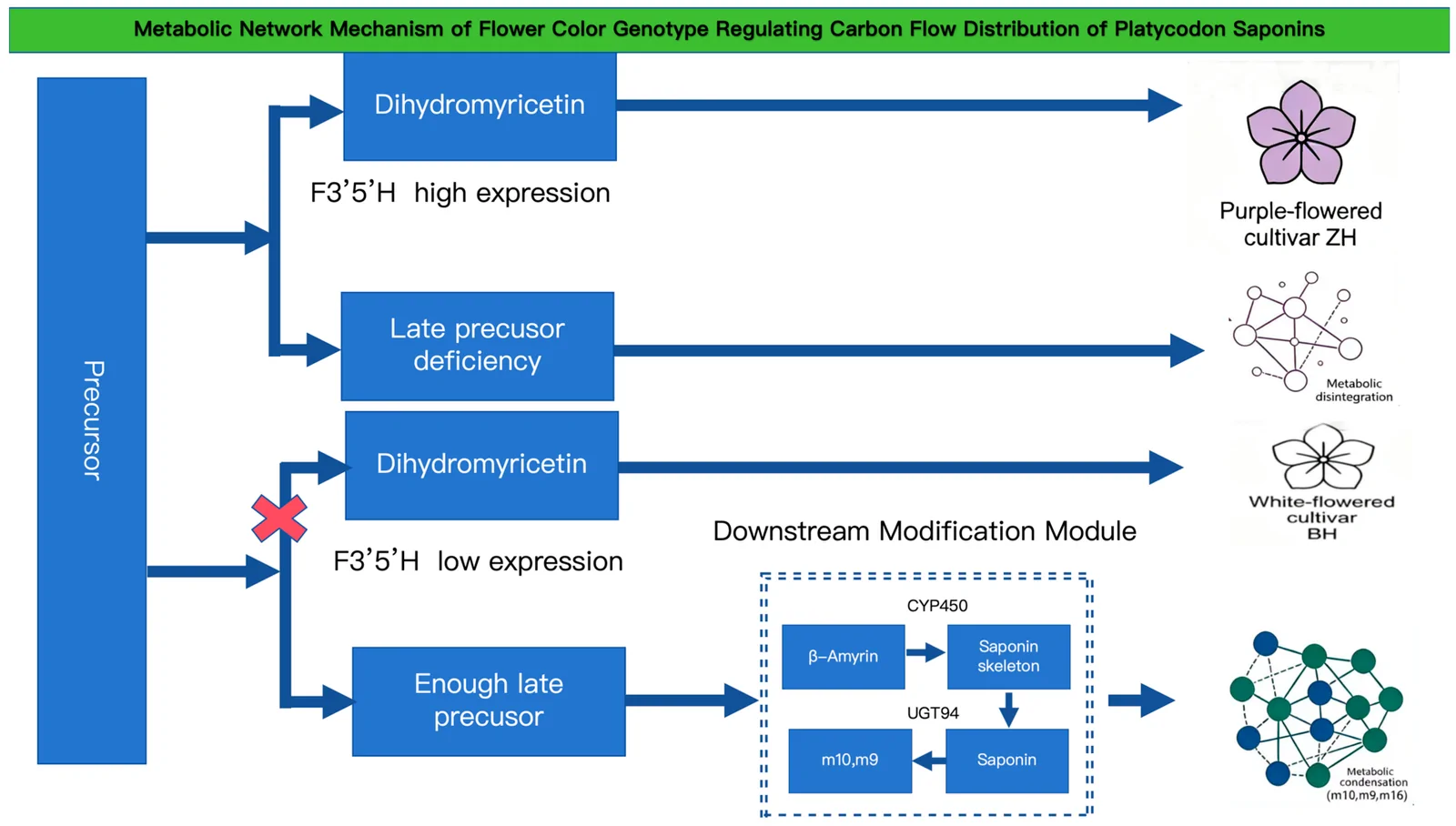
Hypothetical schematic model of flower color genotype-mediated carbon flux redistribution in *Platycodon grandiflorus* reproductive organs. This model is only deduced from untargeted metabolomic data and has not been validated by transcriptomic or functional enzyme experiments. Purple-flowered (ZH): high F3′5′H expression channels precursors to anthocyanins, causing late-stage precursor deficiency and metabolic disarray. White-flowered (BH): silenced F3′5′H blocks anthocyanin synthesis, redirecting flux via the MVA/MEP pathway to enhance UGT94-mediated glycosylation/acetylation, forming a coordinated saponin accumulation module (acetyl-m12, m10, m9, and m16) at the young fruit stage. The timeline depicts the shift from mid-stage substrate competition to late-stage genotype-driven carbon flow differentiation.

Mfuzz time-series clustering, PCA, and dynamic metabolic network topology consistently demonstrate the spatiotemporal differentiation of triterpenoid saponins and flavonoids in *P. grandiflorus* reproductive organs, imposed by carbon flux redistribution in response to F3′5′H functional loss. Figure 5: flowered plants form a tight synergy saponin module at the young fruit stage; purple-flowered plants lose metabolic synergy.

We can conclude that the young fruit period of white-flowered plants is the best harvest period for bioactive saponins, whereas the middle bud to full blooming period of purple-flowered plants is the appropriate period for flavonoid accumulation. Further studies must focus on combining T2T genome data with tissue-specific transcriptomic and functional validation.

## Figures and Tables

**Table 1:** Metabolite clustering results.

Metabolite	Identification	Type of Compound	Cluster
m13	Platycodin L or isomer [20]	Saponin	1
m25	Acetyl-platycodin D	Saponin	1
m11	3″-O-acetylplatycodin D2 or isomer [21]	Saponin	1
m7	Platycoside H	Saponin	1
m19	Platycodin L	Saponin	2
m18	3″-O-Acetyl-ploygalacin D3 or isomer	Saponin	2
m17	Platycodin L or isomer	Saponin	2
m14	3″-O-Acetyl-ploygalacin D3 or isomer	Saponin	2
m5	Platysaponin A	Saponin	2
m20	Acetyl-m12 or isomer	Saponin	3
m16	Acetyl-m12 or isomer	Saponin	3
m12	Unknown (platcodic acid-type saponin)	Saponin	3
m24	Isorhamnetin 3-(2″-O-acetylglucoside)	Flavonoids	4
m23	Isorhamnetin glucoside	Flavonoids	4
m21	Isorhamnetin-3-O-rutinoside	Flavonoids	4
m1	Benzoylmalic acid-O-rhamnosyl-malonyl or isomer	Flavonoids	4
m6	Platycodins J or isomer	Saponin	5
m4	Lobetyolin	Lobetyolin	5
m22	Taxifolin	Flavonoids	5
m15	Platycodins J or isomer	Saponin	6
m10	Platycodin D	Saponin	6
m9	Platycodin D3	Saponin	6
m8	Platycodin L or isomer	Saponin	6
m3	Apigenin-7-O-rutinoside	Flavonoids	6
m2	Luteolin-7-O-rutinoside	Flavonoids	6

## Data Availability

The original contributions presented in this study are included in the article/Supplementary Materials. Further inquiries can be directed to the corresponding author.

## Supplementary Materials

The following supporting information can be downloaded at https://www.mdpi.com/article/10.3390/life16081260/s1, Table S1: Saponins and related secondary metabolites in the reproductive organs of purple-flowered and white-flowered *Platycodon grandiflorus* at six developmental stages were detected by broad-spectrum mass spectrometry.

## Abbreviations

UPLC-MS/MS	Ultra-performance liquid chromatography tandem mass spectrometry
PCA	Principal component analysis
OPLS-DA	Orthogonal partial least squares discriminant analysis
F3′5′H	Flavonoid 3′,5′-hydroxylase
IPP	Isopentenyl pyrophosphate
DMAPP	Dimethylallyl pyrophosphate
MVA	Mevalonate
MEP	2-C-Methyl-D-erythritol 4-phosphate
CYP450	Cytochrome P450
UGT	UDP-glycosyltransferase
